# Age-Associated Differences in Paddock Locomotor Activity Among Senior Horses: A Pilot Observational Study

**DOI:** 10.3390/ani16081208

**Published:** 2026-04-15

**Authors:** Luc Poinsard, Claire Anson, Véronique Billat

**Affiliations:** 1 EA 4445—Movement, Balance, Performance, and Health Laboratory, Université de Pau et des Pays de l’Adour, 65000 Tarbes, France; lpoinsard@univ-pau.fr; 2 Horse Run Impulse Association, 77123 Noisy-sur-Ecole, France; claireanson@yahoo.fr; 3 Faculty of Sport Science, Université Évry Paris-Saclay, 91000 Évry-Courcouronnes, France

**Keywords:** horses, ageing, locomotion, monitoring, welfare

## Abstract

Turnout locomotor activity is a potentially informative indicator of health and welfare in older horses, yet objective field data in seniors remain limited. We examined whether a brief turnout recording could detect cross-sectional associations between chronological age and locomotor activity in senior horses in this study setting. In this single-site observational study, 28 senior Selle Français horses (17–35 years) contributed 122 paddock sessions (2 h each), with total distance and mean speed quantified using a Polar Team Pro sensor. Associations with age were assessed using linear mixed-effects models adjusted for temperature and precipitation. Age was decomposed into between-horse and within-horse components to separate cross-sectional from within-horse information. Raw (untransformed) total distance ranged from 148 m to 3994 m (median 1128 m; IQR 638–1779 m; mean 1292 ± 834 m). Log-transformed total distance was negatively associated with age (β = −0.062 per year; 95% CI −0.094 to −0.032; *p* < 0.001), driven by the between-horse component (β = −0.063; q = 0.003). The within-horse estimate was imprecise and not statistically supported (*p* = 0.75). Mean speed showed a similar pattern, with a significant between-horse association (β = −0.060; q = 0.003) and an imprecise within-horse estimate (*p* = 0.87). These findings suggest that brief paddock actimetry may help characterize between-horse heterogeneity and support group-level welfare monitoring. However, the present dataset does not allow robust inference about within-horse ageing trajectories or individual-level biological ageing. Larger multi-site cohorts with denser follow-up and external validation are needed before individual trajectories or clinical interpretation can be established.

## Simple Summary

As horses age, changes in daily movement may reflect their health and overall well-being. However, there is limited objective information about how normal ageing affects movement in older horses under everyday management conditions. In this study, we investigated whether a short turnout period in a paddock could reveal age-related differences in movement in senior horses. Twenty-eight older sport horses aged 17 to 35 years were monitored during 122 turnout sessions lasting two hours, over a period of approximately two and a half years. Movement was recorded using a small wearable tracking device that measured how far and how fast the horses moved. We found that older horses generally covered less distance and moved more slowly than younger senior horses (e.g., a horse 5 years older was estimated to cover approximately 27% less total distance during the 2 h window). In this dataset, the detectable age-related signal was mainly observed in comparisons between horses. Because repeated follow-up within horses was sparse and unbalanced, within-horse ageing trajectories could not be assessed precisely. These findings suggest that short turnout monitoring may help identify differences in activity levels among older horses and could support welfare monitoring at the group level. Larger multi-site studies are needed before this approach can be used to assess health changes in individual horses.

## 1. Introduction

Ageing in horses has been associated with progressive changes in aerobic capacity and musculoskeletal function. In unfit mares, older horses showed lower maximal heart rate and maximal oxygen consumption than young and middle-aged animals [1], consistent with review evidence indicating a broader decline in aerobic capacity and exercise tolerance with advancing age [2]. Ageing has also been linked to skeletal muscle metabolic changes suggestive of reduced oxidative capacity [3], as well as to differences in muscle fibre composition and body composition in older mares [4]. In addition, altered glucose–insulin dynamics have been reported in aged mares [5]. These findings suggest that ageing may influence not only exercise performance but also the physiological basis of everyday locomotion.

Epidemiological and clinical field studies indicate that geriatric horses carry a substantial burden of chronic abnormalities, with locomotor impairment among the most frequent concerns. Compared with younger horses, geriatric horses have been reported to have more frequent histories of lameness and other age-associated disorders [6]. In horses aged ≥30 years, veterinary examinations identified lameness and reduced joint range of motion far more often than owners reported them, indicating that clinically meaningful mobility limitations may be under-recognized in the field [7]. A larger veterinary clinical assessment similarly found high prevalences of lameness, reduced range of motion, hoof abnormalities, and dental disease in geriatric horses [8], and review evidence supports the broader conclusion that several major body systems are commonly affected in aged horses [9]. Recent work using wearable monitoring in older horses further shows that activity time budgets can be quantified objectively under field conditions, while also highlighting marked effects of housing and turnout conditions on activity profiles [10,11]. These observations support the search for simple, field-deployable metrics able to capture functional differences among older horses under the management conditions of the study site.

Older horses are a heterogeneous population living under highly variable management conditions. Review evidence indicates that regular turnout remains common in geriatric horses, although exercise level, feeding practices, and turnout duration differ across individuals and may change after retirement [6,9]. In human ageing research, wearable accelerometry has been widely used to quantify free-living movement behaviour and to identify movement patterns associated with mortality and healthy longevity [12,13,14]. A comparable question in equine practice is whether unprompted locomotor behaviour recorded during turnout can provide an objective characterization of age-related differences under the management conditions of the study site. Here, unprompted refers to locomotor behaviour expressed during a 2 h individual turnout window without forage provision or environmental enrichment and in the absence of ridden or handler-driven exercise. Because turnout locomotion is shaped not only by intrinsic functional capacity but also by exercise history, paddock size, pasture management, and social context, it should be viewed as a context-sensitive behavioural phenotype rather than a simple proxy of physical capacity [15,16,17].

Under a standardized paddock protocol, wearable sensors offer an objective, scalable approach to quantifying locomotor activity in field conditions. Recent work highlights the growing range of validated technologies for monitoring physiological and behavioural parameters in horses outside laboratory settings [18]. In horses, accelerometry-based systems have been shown to distinguish different activity levels and gaits under controlled conditions [19,20], while field studies have also used sensor-based approaches to quantify activity patterns and time budgets under free-living management conditions [16]. At the same time, available studies indicate that measurement performance depends on the recording context and sensor placement, and evidence specifically targeting short-duration paddock recordings in senior horses remains limited.

In the present pilot study, we evaluated whether a 2 h paddock actimetry protocol can detect cross-sectional, age-associated differences in spontaneous locomotor activity among senior horses under the management conditions of the study site. We modelled associations between age (17–35 years) and GNSS-derived locomotor metrics using mixed-effects models and decomposed age into between-horse and within-horse components.

Based on reported age-associated differences in exercise performance and the high prevalence of chronic musculoskeletal conditions in geriatric horses, we hypothesized that older age at recording would be associated with lower locomotor output during the 2 h paddock window (H1). Given the sparse and unbalanced follow-up, we expected the between-/within-horse decomposition to be more informative for characterizing cross-sectional patterns than within-horse change, while recognizing that within-horse trajectories might not be estimable precisely over the available follow-up (H2).

## 2. Materials and Methods

### 2.1. Study Design and Setting

This observational field study analyzed locomotor activity collected during 2 h paddock recordings between April 2022 and November 2024 at Chambergeot Équitation (Noisy-sur-École, Seine-et-Marne, France). The 2 h recording window was selected as a pragmatic duration compatible with routine yard constraints and repeated measurements across horses. Similar short-duration turnout windows have been used in managed sport-horse settings [21].

### 2.2. Study Population and Cohort Definitions

The dataset comprised 28 Selle Français horses (20 geldings, 8 mares) contributing 122 recordings (1–10 per horse; age range 17–35 years; mean age 23.0 ± 5.03 years). Estimated body mass was derived from morphometric measurements using the equation proposed by Martin-Rosset [22], based on height at withers and heart girth; the mean estimated body mass was 473.8 ± 43.52 kg. A body condition score (BCS) was assessed by visual inspection and palpation of subcutaneous fat deposits using a 0–5 scale (0 = emaciated; 5 = obese) following the French Horse and Riding Institute (IFCE) field protocol for equine body condition scoring [23]. Scoring was performed across six anatomical sites (withers, neck crest, ribs, behind the shoulder, tailhead, and croup), and an overall BCS was calculated as a weighted mean of site scores (withers 0.50, neck crest 0.15, behind the shoulder 0.15, ribs 0.10, tailhead 0.05, croup 0.05). The mean horse-level BCS was 3.1 ± 0.56.

Definitions of “senior”, “aged” or “geriatric” horses vary across studies. In the present study, we operationally defined the senior cohort as horses aged ≥17 years in order to focus on an older segment of the commonly used ageing range and reduce overlap with mid-age mature horses under comparable field conditions [24].

Information on routine health management and chronic medication use was collected from horse owners and yard records. Horses received routine preventive care (e.g., farriery and dental care) according to standard practice at the facility. Based on available owner/yard records, no horse in the analytical sample was reported to have recurrent lameness or to be receiving chronic medication, including long-term analgesics/non-steroidal anti-inflammatory drugs (NSAIDs) or pergolide. In addition, before each recording session, horses underwent routine screening for overt locomotor impairment using EquiSym^®^ (Arioneo, Paris, France). However, health status was not established through a standardized veterinary diagnostic work-up (e.g., systematic lameness examination or endocrine testing for pituitary pars intermedia dysfunction (PPID) and equine metabolic syndrome (EMS)). Therefore, residual confounding due to unrecorded or subclinical comorbidities cannot be excluded.

The number of recordings per horse varied because monitoring was conducted in a real-world, privately owned yard with a dynamic population rather than in a closed cohort. Some horses entered follow-up after becoming eligible, whereas others were no longer followed because they changed yard or, in some cases, died. Among horses leaving follow-up, four died and nine changed yards. Because entry into and exit from follow-up were not prospectively coded in a standardized form in relation to age or clinical status, the possibility of informative attrition cannot be excluded.

A small reference group of younger horses (<17 years; 5 geldings, 2 mares; race: Selle Français) recorded in the same facility under the same 2 h turnout protocol was available and was used for a secondary, contextual comparison only. This group was not used to define the senior cohort and was analyzed descriptively to provide context for locomotor outcomes under the same recording conditions (Table 1).

To improve transparency regarding the repeated-measures structure, we additionally summarize the distribution of recordings per horse in Supplementary Figure S1 and Supplementary Table S1. These materials show the number of sessions contributed by each horse and the corresponding observation span, thereby making the sparse and unbalanced follow-up structure explicit.

### 2.3. Recording Environment and Procedures

Recordings were conducted in four paddocks arranged as two rows of two paddocks separated by a central path. Each paddock measured approximately 50 m × 25 m, was sparsely grassed, and was located on flat, open terrain with an unobstructed sky view. Paddocks contained no shelters or environmental enrichment; no hay or supplementary feed was provided during the 2 h recording window. Each paddock was equipped with a water trough available ad libitum. Horses were kept alone in their paddock. They could see other horses but had no direct contact. To minimize proximity effects and reduce variation in social context, horses were rotated across paddocks, and horses recorded at the same time were placed in opposite corners across the central path.

Recordings were scheduled under stable yard routines and were generally performed in the morning, although they could occasionally extend into the afternoon because of practical constraints. Within each horse, sessions were kept at the same general time of day whenever possible. For a given horse, recording sessions were planned approximately every 3 months, although the interval could occasionally extend to ~4 months due to practical constraints. This resulted in repeated longitudinal observations of the same horse across seasons. Horses were already accustomed to the paddocks used for recording.

Outside the recording sessions, horse management was not standardized and depended on owners and routine yard practice. Based on yard-level information, ridden exercise was generally of approximately 1 h and usually took place in the surrounding Fontainebleau forest environment, with a large proportion of walking (>75%) and smaller amounts of trot and canter. However, the exact type, intensity, and duration of work varied between horses and were not prospectively recorded in a standardized way. Some horses were likely ridden mostly at walk, whereas others performed more trot or canter. In general, horses had daily access to paddock turnout when they were not ridden, typically for approximately 2 h.

To reduce confounding from structured exercise, horses were not worked the day before recording. If horses were exercised on the day before recording, this was typically limited in routine practice to low-intensity activity, usually consisting predominantly of walking with only small amounts of trot or canter. However, this was not quantified using a standardized scale and reflected routine rider/yard judgement rather than formal activity control. Prior to each paddock recording, horses underwent routine screening for overt locomotor compromise using (i) an EquiSym^®^ (Arioneo, Paris, France) assessment aimed at detecting marked locomotor asymmetry [25,26], and (ii) a welfare-oriented evaluation using the IFCE “Cheval Bien-Être” application (version 1.3), an adaptation of the AWIN Horse protocol based on animal- and resource-based indicators across four welfare principles (feeding, housing, health, and behaviour) and intended for welfare appraisal rather than veterinary diagnosis [27,28]. For EquiSym^®^ screening, each horse was trotted on two surfaces (firm and soft) under three conditions: a 30 m straight-line out-and-back, five circles to the left, and five circles to the right. EquiSym^®^ outputs (vertical displacement curves and derived asymmetry indicators) were reviewed by a veterinarian to screen for marked left–right asymmetry at the time of recording. Because EquiSym^®^ is a decision-support tool and does not provide a single universally applicable numerical cut-off for exclusion, screening relied on the veterinarian’s interpretation of the device-generated indicators within the standardized trot conditions described above. These procedures were used to document clinical status and to flag sessions potentially affected by acute impairment. No sessions were judged to show marked asymmetry consistent with overt lameness, and therefore no recordings were excluded based on EquiSym^®^ or welfare evaluations.

### 2.4. Instrumentation and Outcomes

Locomotor activity and displacement were recorded using Polar Team Pro (Polar Electro, Kempele, Finland) with 10 Hz GNSS sampling. The sensor was clipped into a Polar belt fitted externally around the horse’s thorax during the 2 h recording session. Polar Team Pro has shown acceptable validity and reliability for distance- and speed-related metrics across controlled running conditions, with performance depending on setting (e.g., indoor vs. outdoor) and movement pattern [29,30]. Although equine-specific validation of this device in paddock conditions is not available, GPS-based monitoring has been applied in horses in pasture settings [31]. Given the open-sky paddock environment and plausibility-based QC, GNSS-derived total distance and mean speed were used as session-level indicators of turnout locomotor activity in this exploratory study.

The sensor was started at the beginning of each 2 h recording and stored GNSS-derived data. At the end of the session, the sensor was retrieved and recordings were uploaded to the Polar platform, from which session data were exported in spreadsheet format for analysis.

The primary outcome was total distance covered during the 2 h window, derived from GNSS displacement. A secondary locomotor outcome was mean speed over the same window.

### 2.5. Meteorological Covariates

Hourly temperature and precipitation totals (mm) were obtained from Météo-France (nearest station: Fontainebleau, Seine-et-Marne, France) and aggregated over each 2 h recording window using overlap-weighted summaries (temperature: mean; precipitation: sum), based on session start times extracted from the Polar platform.

### 2.6. Ethics Statement (France)

The study involved non-invasive field monitoring only and did not include procedures expected to cause pain, suffering, distress, or lasting harm. Owners provided informed consent. Under French regulations governing the use of animals for scientific purposes (Code rural et de la Pêche maritime, Articles R214-88 and R214-89) [32,33], activities not liable to cause pain, suffering, distress, or lasting harm equivalent to or greater than that caused by the introduction of a needle (good veterinary practice) fall outside the scope of regulated experimental procedures; accordingly, no formal project authorization/ethical review was sought for this observational monitoring.

### 2.7. Data Processing and Quality Control

Quality control additionally targeted GNSS artefacts that can disproportionately affect low-speed movement. Recordings were visually screened for unrealistic displacement spikes and non-physiological speeds. We applied rule-based plausibility checks at the epoch level (10 Hz), including removal of observations with instantaneous speed > 15 m·s^−1^ and/or point-to-point displacement >5 m between consecutive samples (0.1 s), before computing session-level totals. Sessions with substantial GNSS signal loss (>20% missing/invalid epochs) or erratic traces were excluded from GNSS-derived analyses. Because low-speed GNSS can show higher relative error, analyses focused on aggregated 2 h session metrics obtained under an open-sky paddock set-up.

These QC steps were implemented a priori. No sessions exceeded the predefined GNSS exclusion thresholds in this dataset.

### 2.8. Statistical Analysis

Analyses were performed in RStudio (version 2026.01.0+392) using lme4 for model fitting, lmerTest for inference, and performance for diagnostic checks and model summaries [34,35,36].

All analyses were conducted at the recording level while accounting for repeated measurements within horses. Because multiple 2 h recordings were available for some horses (1–10 per horse), observations were not independent. Treating each recording as an independent data point would risk pseudo-replication and overly optimistic standard errors [37]. Therefore, associations between age at recording and locomotor outcomes were evaluated using mixed-effects models (multilevel/hierarchical models) including a random intercept for horse to model within-horse clustering and account for the unbalanced follow-up structure [38].

#### 2.8.1. Primary Models (Distance and Speed)

For total distance covered during the 2 h paddock window, diagnostics plots from preliminary natural-scale mixed models (residuals versus fitted values, Q–Q plots, and scale–location plots) indicated right-skew and heteroscedasticity, whereas log-transformation improved variance stabilization and overall residual diagnostics. Total distance was therefore analyzed on the log scale using a linear mixed-effects model:

(1) $$\log\left({distance}_{ij}\right)=\beta_{0}+\;\beta_{1}\;{age}_{ij}+\;\beta_{2}\;{temperature}_{ij}+\;\beta_{3}\;{precipitation}_{ij}+\;u_{0i}+\;\epsilon_{ij}$$

where i denotes the horse and j the recording session; ${distance}_{ij}$ denotes the 2 h total distance recorded for horse i at session j; $\beta_{0}$ is the population-level intercept; $\beta_{1}$ is the fixed effect of age at recording; $\beta_{2}$ is the fixed effect of temperature; $\beta_{3}$ is the fixed effect of precipitation; $u_{0i}$ is the deviation of horse i from the population intercept (random intercept); and $\varepsilon_{ij}$ is the session-level residual error. Mean speed was analyzed using an analogous mixed-effects model, as diagnostic checks similarly supported improved model fit on the log scale.

#### 2.8.2. Between-Horse vs. Within-Horse Age Decomposition

Because age varied across recording dates within some horses, age was decomposed into a between-horse component (horse-specific mean age across recordings) and a within-horse component (deviation from the horse mean) to separate cross-sectional from within-horse information:

(2) $${age}_{ij}=\bar{{age}_{i}}+({age}_{ij}-\;\bar{{age}_{i}})$$

Models were refitted with both terms to separate cross-sectional from within-horse information and to describe the relative contribution of each component.

#### 2.8.3. Effect Modification (Age × Season; Age × Sex)

Potential effect modification by season and sex was explored a priori because (i) season may capture broad, unmeasured contextual influences on turnout behaviour beyond contemporaneous weather covariates (e.g., photoperiod and management-related seasonal routines), and (ii) sex differences in locomotor behaviour have been reported in managed horses. To limit multiple testing in this modest sample, we did not systematically screen interactions with additional horse-level covariates (e.g., BCS or estimated body mass) or paddock identity. Paddock effects were minimized by rotating horses across paddocks within a fixed, uniform paddock layout. Interaction models were evaluated using likelihood-ratio tests (ML fits); multiplicity across the four prespecified interaction likelihood-ratio tests (LRTs) (distance × season, speed × season, distance × sex, and speed × sex) was controlled using Benjamini–Hochberg false discovery rate (FDR), with q-values reported [39].

#### 2.8.4. Sensitivity Analyses and Model Reporting

For log-scale models, fixed-effect estimates are reported as β coefficients with 95% confidence intervals; where interpretation is provided, exp(β) is used to express multiplicative change per 1-year increase (percent difference on the original scale). For distance, a Gamma mixed model with a log link was fitted as a distributional sensitivity analysis for strictly positive outcomes. We retained the log-linear mixed model as the primary specification because it showed satisfactory diagnostic behaviour on the transformed scale and provided straightforward interpretation and comparability across outcomes; the Gamma model yielded consistent age estimates. *p*-values for linear mixed models were obtained using Satterthwaite’s degrees-of-freedom approximation [34]. To control multiplicity across endpoints, *p*-values for the age effects (between-horse and within-horse components) across the two outcomes (total distance and mean speed) were adjusted using the FDR procedure, and FDR-adjusted q-values are reported alongside raw *p*-values. Marginal and conditional R^2^ values were reported for mixed models to summarize variance explained by fixed effects alone and by the full model including random effects [40].

As an exploratory sensitivity analysis, the primary mixed-effects models were repeated with BCS added as an additional fixed-effect covariate.

#### 2.8.5. Secondary Contextual Comparison (Young vs. Senior)

In addition to the senior cohort, a small reference group of younger horses (<17 years) was monitored in the same facility under the same recording protocol. As a secondary, contextual analysis, we compared locomotor outcomes between younger and senior horses using mixed-effects models with a random intercept for horse and adjustment for season, sex, and weather (temperature and precipitation). This comparison was intended to provide descriptive context for the magnitude of locomotor outcomes under the same recording protocol and was not included in the multiplicity correction applied to the primary age-effect hypotheses.

## 3. Results

A total of 122 recordings from 28 senior horses were included in the primary analyses. The number of recordings per horse ranged from 1 to 10, confirming a sparse and unbalanced repeated-measures structure (Supplementary Figure S1; Supplementary Table S1).

### 3.1. Total Distance Covered During the 2 h Paddock Window

Across the 122 senior-horse recordings included in the primary analyses, raw total distance over 2 h ranged from 148 m to 3994 m (median 1128 m; IQR 638–1779 m; mean 1292 ± 834 m).

In mixed-effects models accounting for repeated recordings (random intercept for horse), log-transformed total distance covered during the 2 h paddock window decreased with age at the session (β = −0.062 per year, 95% CI −0.093 to −0.031; *p* < 0.001). Temperature (β = 0.011 per °C, 95% CI −0.005 to 0.028; *p* = 0.19) and precipitation (β ≈ 0.0004 per mm, 95% CI −0.039 to 0.040; *p* = 0.98) were not associated with log-distance. The intraclass correlation coefficient (ICC) for log-distance was 0.178 (adjusted).

**Figure 1:**
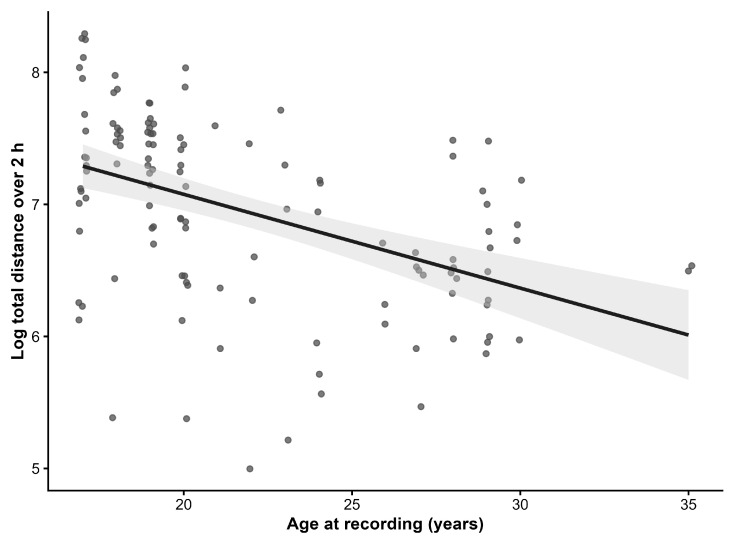
Relationship between age at recording and total distance covered during the 2 h paddock window. Scatterplot of 122 2 h recordings from 28 senior horses. For visualization, the total distance is shown on the natural log scale. The fitted line is a simple linear regression with 95% confidence band. Inferential analyses were performed using mixed-effects models with a random intercept for horse to account for repeated recordings.

Figure 1 displays the raw relationship between age at recording and total distance covered during the 2 h paddock window.

To separate cross-sectional from within-horse information, age was decomposed into between-horse and within-horse components. The between-horse component was negatively associated with log-distance (β = −0.063 per year, 95% CI −0.094 to −0.032; *p* < 0.001; FDR q = 0.003). The within-horse estimate was also negative in direction but highly imprecise (β = −0.031 per year, 95% CI −0.217 to 0.155; *p* = 0.75; FDR q = 0.872).

As a sensitivity analysis, a Gamma mixed-effects model with a log link yielded consistent results, also showing a negative association between age and total distance (β = −0.058 per year; 95% CI −0.096 to −0.020; *p* = 0.003).

For the log-distance model, fixed effects explained approximately 20% of the variance (marginal R^2^ = 0.20), while including the horse-level random intercept increased explained variance to 34% (conditional R^2^ = 0.34), indicating both between-horse variability and residual within-horse variability under field conditions.

### 3.2. Mean Speed During the 2 h Paddock Window

Mean speed showed a similar pattern. In models fitted on the log scale to improve residual diagnostics, the between-horse age component was negatively associated with log(mean speed) (β = −0.060 per year, 95% CI −0.094 to −0.027; *p* = 0.00169; FDR q = 0.003). The within-horse estimate was again imprecise and not statistically supported (β = −0.016 per year, 95% CI −0.209 to 0.177; *p* = 0.87; FDR q = 0.872). Temperature (β = 0.011 per °C, 95% CI −0.006 to 0.028; *p* = 0.22) and precipitation (β = −0.005 per mm, 95% CI −0.046 to 0.036; *p* = 0.81) were not associated with log(mean speed). The ICC for log-speed was 0.192 (adjusted).

**Figure 2:**
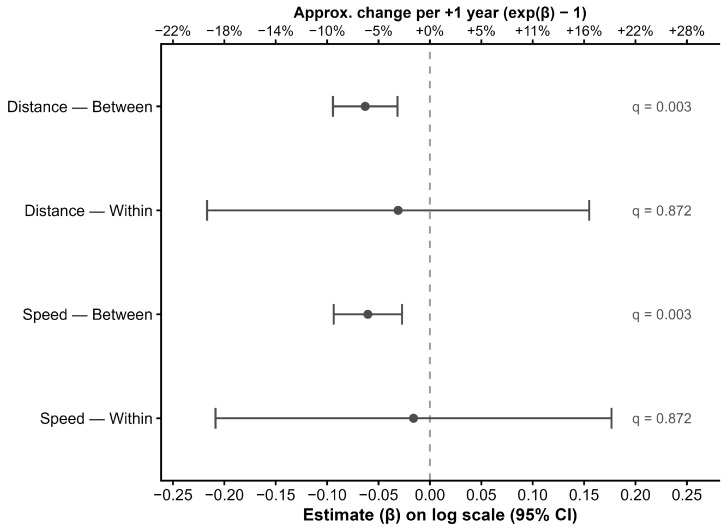
Between-horse and within-horse age effects on locomotor outcomes. Fixed-effect estimates (β) and 95% confidence intervals from mixed-effects models with random intercept for horse, after decomposing age into between-horse (horse mean age across recordings) and within-horse (deviation from horse mean) components. Outcomes are modelled on the log scale; exp(β) gives the multiplicative ratio associated with a 1-year difference in age. FDR-adjusted q-values (Benjamini–Hochberg) are reported for the age terms. For example, β = −0.063 corresponds to exp(−0.063) ≈ 0.94, i.e., an estimated ~6% lower total distance for a horse that is 1 year older (between-horse component), holding covariates constant. Over a 5-year difference, the expected ratio is exp(5 × β) = exp(−0.315) ≈ 0.73, corresponding to ~27% lower distance for a horse that is 5 years older (e.g., 25 vs 20 years), under the model’s assumption of a constant multiplicative change per year.

Age effects for distance and mean speed are summarized in Figure 2 (between- and within-horse components; log scale), with 95% confidence intervals and BH-FDR-adjusted q-values.

### 3.3. Exploratory Interaction and Sensitivity Analyses

Exploratory interaction analyses provided no evidence that adding age × season interactions improved fit for total distance (LRT χ^2^(9) = 11.42, *p* = 0.248; BH-FDR q = 0.331) or mean speed (LRT χ^2^(9) = 9.26, *p* = 0.414; BH-FDR q = 0.414). Similarly, age × sex interaction models did not improve fit for distance (LRT χ^2^(2) = 4.47, *p* = 0.107; BH-FDR q = 0.214) or speed (LRT χ^2^(2) = 4.77, *p* = 0.092; BH-FDR q = 0.214). Type III Wald tests suggested a nominal age_mean_id × sex term for speed (*p* = 0.047) (and a trend for distance, *p* = 0.057), but these were not supported by the global LRTs and were not interpreted as confirmatory.

In an exploratory sensitivity analysis, additional adjustment for BCS did not materially alter the main findings. The between-horse age association remained negative for both log-total distance (β = −0.071, *p* < 0.001) and log-mean speed (β = −0.065, *p* < 0.001), whereas the within-horse age component remained imprecisely estimated and was not statistically supported. BCS itself was not significantly associated with either outcome (distance: β = 0.087, *p* = 0.422; speed: β = 0.082, *p* = 0.476).

As an additional sensitivity analysis, the between-/within-horse models were repeated in the subset of horses contributing at least three recordings (110 recordings from 19 horses). The pattern of results was similar to the primary analysis. For log-total distance, the between-horse age component remained negative (β = −0.069, 95% CI −0.108 to −0.029; *p* = 0.002), whereas the within-horse estimate remained imprecise and not statistically supported (β = −0.033, 95% CI −0.222 to 0.156; *p* = 0.731). Mean speed showed a similar pattern, with a negative between-horse age association (β = −0.065, 95% CI −0.107 to −0.024; *p* = 0.004) and an imprecise within-horse estimate (β = −0.017, 95% CI −0.213 to 0.179; *p* = 0.860).

### 3.4. Secondary Contextual Comparison (Younger vs. Senior Horses)

To provide a contextual reference for the magnitude of locomotor outcomes, we additionally analyzed a small group of younger horses (see Table 1) recorded in the same facility under the same 2 h turnout protocol. In mixed-effects models with a random intercept for horse and adjustment for season, sex, temperature, and precipitation, senior horses (≥17 years) showed lower locomotor activity than younger horses, with lower log-total distance (β = −0.570, 95% CI −0.930 to −0.211; *p* = 0.005) and lower log-mean speed (β = −0.556, 95% CI −0.921 to −0.192; *p* = 0.007). On the log scale, these estimates correspond to 44% lower total distance (exp(β) = 0.565) and 43% lower mean speed (exp(β) = 0.573) in seniors compared with younger horses. The ICCs for horse-level clustering were 0.318 (unadjusted) and 0.304 (adjusted) for log-distance, and 0.304 (unadjusted) and 0.293 (adjusted) for log-speed.

## 4. Discussion

This single-site observational field study suggests that a 2 h turnout recording protocol can detect cross-sectional associations between chronological age and GNSS-derived turnout activity metrics among senior horses under routine field conditions at this facility. In mixed-effects models accounting for repeated measures, both total distance and mean speed were negatively associated with age after adjustment for meteorological covariates. When age was decomposed into between-horse and within-horse components, the detectable signal in this dataset was concentrated in the between-horse component, whereas within-horse estimates were too imprecise to support meaningful inference about individual ageing trajectories. A small, non-matched reference group of younger horses recorded under the same protocol showed higher locomotor metrics; this secondary comparison is reported for contextual description only.

### 4.1. Interpretation of Cross-Sectional Associations Between Age and Paddock Locomotion

The observed cross-sectional association between age and lower turnout activity may reflect multiple, non-mutually exclusive factors. Field studies report a high burden of chronic conditions in geriatric horse populations, including musculoskeletal abnormalities [7,8], and age-related changes in muscle properties have been described [3,4]. However, because we did not perform standardized clinical phenotyping, these literature-based factors cannot be evaluated in the present dataset and should be considered only as potential contributors.

Our study did not include standardized clinical examinations, diagnostic imaging, or systematic comorbidity assessment, and therefore cannot determine whether the observed age associations reflect intrinsic biological ageing, accumulated pathology (whether diagnosed or subclinical), cohort effects, or selective survival of less active individuals. The cross-sectional design further precludes inference about within-individual change over time. Other possible explanations include differences in lifetime athletic histories or management backgrounds, survivor bias and unmeasured factors such as hoof quality, dental status, metabolic or endocrine conditions (e.g., PPID, EMS), or chronic low-grade pain.

Paddock actimetry captures self-selected locomotion during turnout under field conditions, rather than maximal performance capacity, and may therefore be sensitive to subtle, multifactorial constraints on voluntary movement. Determining the clinical correlates and drivers of lower turnout activity with age will require prospective studies combining repeated turnout recordings with standardized clinical phenotyping and richer contextual covariate capture. Such studies would help test the hypotheses generated by the present cross-sectional observations.

### 4.2. Interpretation of Between-Horse and Within-Horse Age Components

The absence of a statistically supported within-horse age effect in this study should not be interpreted as evidence that individual horses do not change over time. Rather, the wide confidence intervals around the within-horse estimates indicate limited information for estimating such trajectories in the present dataset [41]. In mixed-effects frameworks, within-horse effects are identified from deviations around each horse’s own mean trajectory and therefore require sufficient repeated observations and within-subject age variation to be estimated precisely [41,42]. Here, follow-up was sparse and unbalanced (1–10 recordings per horse), with several horses contributing only one or two sessions and recordings separated by months. Under these conditions, within-horse estimates are especially vulnerable to measurement noise, short-term behavioural variability, and real-world attrition [41].

In addition, turnout movement behaviour is influenced not only by the horse’s functional state but also by session-level contextual factors such as paddock surface and condition, weather, forage availability, and social context. These factors may vary across sessions and can increase behavioural variability, thereby reducing sensitivity to gradual functional decline under field conditions. Equine time-budget and sensor-based studies report substantial variation in locomotion and movement-related behaviours across farms and management systems, indicating that husbandry and environment can contribute importantly to observed activity differences [10,11]. Accordingly, the present results are more appropriately interpreted as showing a cross-sectional association detectable at the between-horse level within this dataset, rather than demonstrating that ageing effects are primarily or exclusively between horses. The observed between-horse association may reflect several non-exclusive processes, including ageing-related functional differences, baseline heterogeneity, unmeasured health-related confounding, or selection effects linked to survival and retention in follow-up. Detecting within-horse ageing trajectories will require denser repeated measurements, longer observation horizons, and richer clinical and contextual covariate capture.

### 4.3. Effect Modification by Season and Sex

Effect modification by season and sex was examined exploratorily because turnout behaviour is context-dependent, and seasonal and management factors are known to influence equine activity time budgets and movement patterns in field settings [10,11]. Interaction models provided no supported evidence that the age association differed by season or sex. Although a nominal age × sex term was suggested by a Wald test for speed, it was not supported by the corresponding global interaction test and was therefore not interpreted as confirmatory. Power to detect effect modification was limited by the temporal sampling scheme, with approximately one observation per season per horse in many cases. Denser repeated recordings would be required to characterize within-horse seasonal variability and improve sensitivity to interaction effects.

### 4.4. Variability Under Field Conditions and Implications for Measurement Strategy

The mixed-model R^2^ values support this interpretation. Fixed effects explained about one-fifth of the variance in log-distance (marginal R^2^ = 0.20), whereas including the horse-level random intercept increased explained variance to 0.34 (conditional R^2^ = 0.34), highlighting substantial between-horse variability alongside considerable residual variability at the session level. These results reinforce two practical implications for field use. First, repeated measurements within individuals are likely to be more informative than single cross-sectional observations when the outcome is behaviorally driven and subject to high day-to-day variability. Second, recording contextual covariates (e.g., paddock surface/condition, feeding timing, social context, and local weather during the 2 h recording window) may improve statistical adjustment and reduce unexplained variance. The absence of detectable effects of temperature and precipitation should be interpreted cautiously, because station-derived measures—even when aggregated over the 2 h window—may not fully capture paddock micro-conditions experienced by the horse (e.g., wind exposure, solar radiation, localized surface moisture), and measurement error can attenuate associations [43,44]. In addition, the limited ranges of weather conditions within a single facility or seasonally clustered sampling can further constrain the power to detect modest meteorological effects on spontaneous locomotion.

### 4.5. Implications and Future Directions

From a practical perspective, these findings support the feasibility of short-duration field actimetry as an objective readout of functional variation among older horses in this field setting. In this dataset, however, the sampling structure and field variability limited the ability to resolve within-horse trajectories, highlighting the need for future work with stronger longitudinal inference.

Larger multi-site cohorts would improve generalizability, increase heterogeneity in management and environmental contexts, and enable explicit modelling of site-level effects. Denser repeated measurements within horses would increase precision for estimating within-horse change and help distinguish gradual decline from short-term behavioural fluctuation.

Longer monitoring windows (e.g., multi-day recordings) would better capture daily rhythms and time-budget structure, providing more stable individual activity profiles than a single 2 h snapshot. Future work should also include finer logging of recording conditions, such as ground/surface state, forage availability during the 2 h period, and notable behavioural events, as these factors may meaningfully influence turnout locomotion and inflate within-horse variance.

Recent scoping work highlights the growing use of wearable and remote monitoring technologies for equine behaviour and health, supporting the relevance of developing field-applicable digital phenotypes in horses [45]. Beyond total distance and mean speed, extracting richer activity features that may be less context-sensitive, such as bout structure, inactivity time, and within-session variability or fragmentation, could improve sensitivity to functional differences and enhance individual stability. Short-term repeatability should be quantified in a subset of horses using two to three closely spaced sessions to characterize measurement noise and inform the number of repeated recordings required for reliable monitoring.

Finally, systematic recording of comorbidities and medications, hoof/farriery status, and recent laminitis history will be important because these factors may influence locomotor output and contribute to unexplained within-horse variability. Linking actimetry-derived outcomes to independent clinical endpoints, such as lameness or pain scores, endocrine status, muscle condition, and owner-reported quality of life, will also be important for validating the clinical meaning of reduced turnout locomotion and for developing multidimensional geriatric monitoring approaches. In this context, activity-derived digital phenotypes may ultimately contribute to functional ageing indices, but such applications will require prospective calibration and external validation.

### 4.6. Limitations

These findings should be interpreted considering several design, measurement, and clinical characterization limitations. Indeed, external validity is limited because recordings were collected in a single private yard, which may restrict generalizability to other management systems and environmental contexts. The sample size was modest (28 horses; 122 recordings), and follow-up was unbalanced (1–10 recordings per horse). Although mixed-effects modelling appropriately accounted for repeated measures and unequal numbers of observations, the available follow-up remained limited for estimating within-horse age-related change. This reflected real-world attrition and management changes, including relocation, ownership changes, and, in some cases, death or euthanasia in older horses, and may also have introduced informative missingness that could not be formally quantified.

Residual confounding by health status also remains possible because horse-level clinical phenotyping was not standardized (e.g., systematic lameness examination, imaging, endocrine/metabolic screening, and comprehensive medication documentation), including no standardized veterinary diagnostic work-up for PPID or EMS. This is particularly relevant in senior horses, in whom subclinical comorbidities may influence spontaneous turnout locomotor activity. Body condition score may also be relevant when interpreting locomotor heterogeneity in senior horses, as it can reflect aspects of health status, nutritional state, or endocrine/metabolic dysregulation. In an exploratory sensitivity analysis, additional adjustment for BCS did not materially change the estimated between-horse age associations for either total distance or mean speed, and BCS itself was not statistically associated with these outcomes. However, this analysis was exploratory and should therefore be interpreted cautiously rather than definitively.

In addition, session-level contextual determinants of turnout locomotion (e.g., footing/substrate conditions, paddock micro-conditions, and salient behavioural events during the 2 h window) were not fully quantified, and station-derived weather covariates may incompletely capture paddock conditions during the specific recording period. Device validity represents another limitation. Polar Team Pro GNSS metrics have been validated primarily in humans, and equine-specific validation under paddock conditions is lacking. Measurement error, particularly at low speeds, may therefore have attenuated associations, despite plausibility-based quality control and an open-sky recording set-up. Finally, the 2 h recording window provides only a partial snapshot of turnout behaviour, and short-term test–retest reliability of this window was not assessed.

## 5. Conclusions

Despite these limitations, the present findings support the feasibility of short-duration paddock actimetry as a low-burden field approach for describing locomotor activity heterogeneity in senior horses. In this single-site observational study, a 2 h paddock recording detected cross-sectional associations between chronological age and GNSS-derived turnout activity metrics under the site-specific management conditions of the study facility. Across 122 recordings from 28 horses aged 17–35 years, older age at recording was associated with lower total distance and mean speed after accounting for repeated measures and meteorological covariates.

However, the present study does not support robust inference about within-horse ageing trajectories. The between-/within-horse decomposition indicates that the detectable age-related signal was concentrated in between-horse comparisons, whereas within-horse estimates were too imprecise to be informative. These results should therefore be interpreted as evidence of cross-sectional locomotor differences associated with age in this cohort, rather than as proof of individual longitudinal decline or biological ageing effects per se.

Larger multi-site cohorts with denser longitudinal follow-up, standardized clinical phenotyping, and external welfare/clinical validation are needed before paddock actimetry can be interpreted as an individual monitoring tool for functional ageing in senior horses.

## Figures and Tables

**Table 1:** Study population/descriptive characteristics.

Group	Horses (n)	Recordings (n)	Age (years)	EBW (kg)	BCS	SPH
Senior (≥17)	28	122	23.0 ± 5.03	471.7 ± 47.82	3.1 ± 0.56	4.4 ± 3.01
Younger (<17)	7	71	13.7 ± 3.45	479.6 ± 32.16	3.4 ± 0.30	5.5 ± 2.96

Note: EBW = estimated body weight (kg); BCS = body condition score; SPH = sessions per horse. Values are presented as mean ± standard deviation.

## Data Availability

The data presented in this study are available on request from the corresponding author.

## Supplementary Materials

The following supporting information can be downloaded at: https://www.mdpi.com/article/10.3390/ani16081208/s1. Figure S1: Number of paddock recording sessions contributed by each horse in the senior cohort; Table S1: Distribution of repeated paddock recordings in the senior cohort.
