# Supplementary material for: Age-Associated Differences in Paddock Locomotor Activity Among Senior Horses: A Pilot Observational Study
Source: Animals (Basel). 2026 Apr 15;16(8):1208. doi: 10.3390/ani16081208 (PMC13113554; doi:10.3390/ani16081208)
Supplement: Supplementary file 1 [file animals-16-01208-s001.zip › animals-4206590-supplementary.pdf]

Table S1. Distribution of repeated paddock recordings in the senior cohort.

Horse_id	Sex	Recordings (n)	Age at first recording (years)	Age at last recording (years)	Observation span (years)	Mean interval between recordings (days)
3	Gelding	10	27	29	2.13	86.6
4	Gelding	5	24	24	0.68	62
5	Gelding	5	22	23	0.68	62
6	Gelding	10	19	21	2.26	91.7
7	Mare	10	18	20	2.14	86.7
8	Gelding	10	17	19	2.20	89.1
9	Mare	7	17	19	2.29	139.3
10	Gelding	6	17	18	1.25	91.4
11	Mare	1	18	18	0	-
12	Mare	5	17	18	1.01	92.2
14	Gelding	4	19	20	1.01	123.3
17	Gelding	5	29	30	0.8	73.2
18	Gelding	4	20	20	0.8	97.7
21	Gelding	1	17	17	0	-
22	Gelding	1	17	17	0	-
23	Gelding	2	35	35	0.01	2
24	Mare	1	27	27	0	-
25	Gelding	2	27	27	0.01	2
26	Gelding	3	28	29	0.7	127.5
27	Gelding	3	30	30	0.61	111.5
28	Mare	3	21	22	0.75	137
29	Gelding	1	18	18	0	-
30	Gelding	3	23	24	0.75	137
31	Mare	1	26	26	0	-
32	Mare	2	26	26	0.01	2
33	Gelding	4	17	17	0.75	91
34	Gelding	5	28	29	1.18	107.5
35	Gelding	8	19	20	1.48	77.4

Note: Within the senior cohort, the number of recordings per horse was not clearly associated with age at first recording (Spearman's rho = -0.162, p = 0.411).

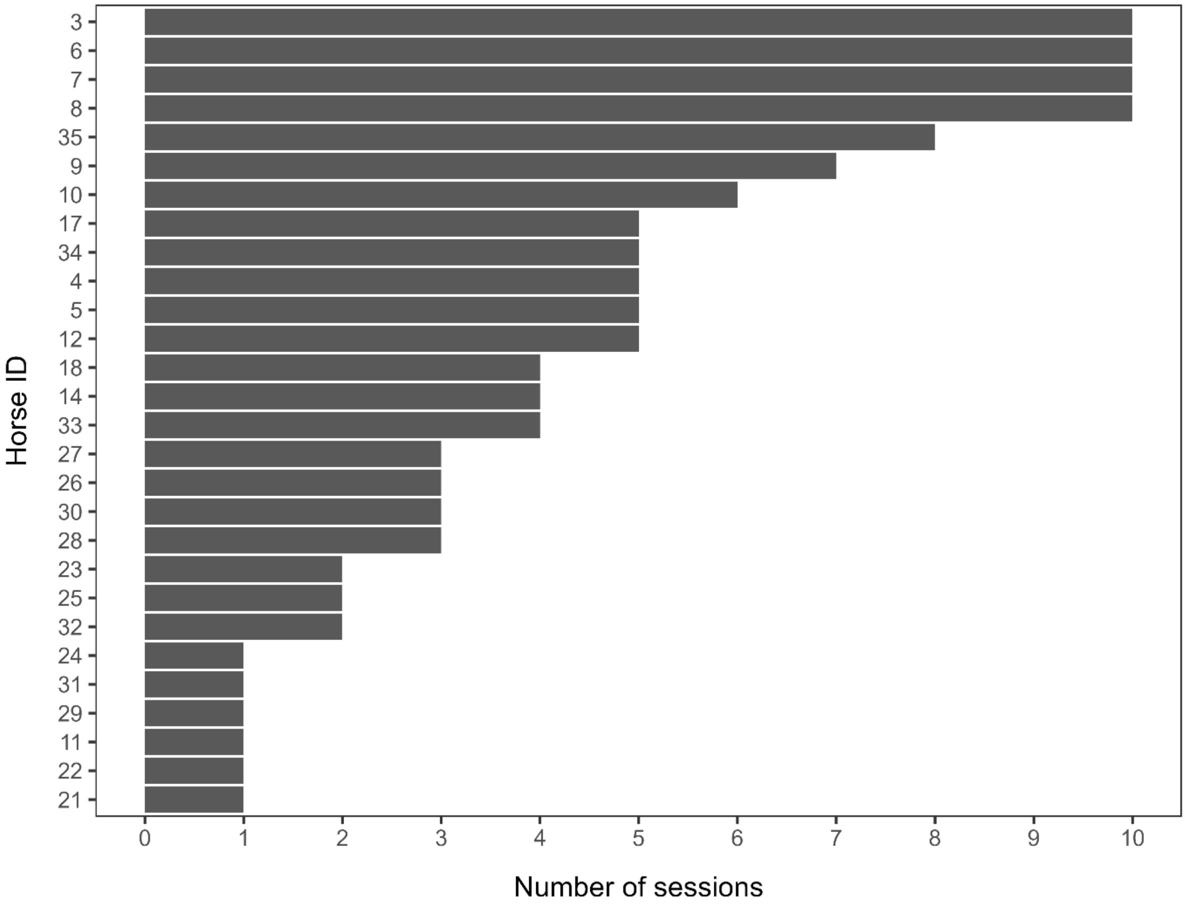

Figure S1. Number of paddock recording sessions contributed by each horse in the senior cohort. The senior cohort comprised 28 horses contributing 122 recordings in total. The number of recordings per horse ranged from 1 to 10, illustrating the sparse and unbalanced repeated-measures structure of the dataset. Bars are ordered by number of sessions contributed per horse.
